# Miniature Noninvasive Sensor Based on Impedance-Change Detection in Branches for Measuring Branch Ice Content in Overwintering Woody Plants

**DOI:** 10.3390/mi14020440

**Published:** 2023-02-12

**Authors:** Hao Tian, Chao Gao, Tao Xie, Chongchong Yu

**Affiliations:** 1 School of Artificial Intelligence, Beijing Technology and Business University, Beijing 100048, China; 2 China Light Industry Key Laboratory of Industrial Internet and Big Data, Beijing Technology and Business University, Beijing 102448, China

**Keywords:** branch volume ice content, edge electromagnetic field induction, impedance measurement, cold stress, miniature sensor, performance analysis

## Abstract

Advancements in detection instruments have enabled the real-time acquisition of water information during plant growth; however, the real-time monitoring of freeze–thaw information during plant overwintering remains a challenge. Based on the relationship between the change in the water–ice ratio and branch impedance during freezing, a miniature noninvasive branch volume ice content (BVIC) sensor was developed for monitoring real-time changes in volumetric ice content and the ice freeze-thaw rate of woody plant branches during the overwintering period. The results of the performance analysis of the impedance measurement circuit show that the circuit has a lateral sensitivity range, measurement range, resolution, measurement accuracy, and power consumption of 0–35 mm, 0–100%, 0.05%, ±1.76%, and 0.25 W, respectively. The dynamic response time was 0.296 s. The maximum allowable error by the output voltage fluctuation, owing to the ambient temperature and humidity, was only ±0.635%, which meets the actual use requirements. The calibration curve fit coefficients were >0.98, indicating a significant correlation. The ice content of plant branches under cold stress was measured for indoor and field environments, and the sensors could effectively monitor changes in the branch ice content in plants exposed to cold stress. Additionally, they can differentiate between plants with different cold resistances, indicating the reliability of the BVIC sensor.

## 1. Introduction

Cold stress is an atypical biotic stress that woody plants face in temperate and cold climates, and is an important factor effecting plant growth and distribution [1,2]. It involves multiple tolerance and avoidance mechanisms in plants, including diffusion through nuclear supercooling, extracellular ice, changes in membrane fluidity, and osmoregulation through ion migration. Cold stress experienced by overwintering plants is essentially a process of the interconversion of liquid water and solid ice as the temperature fluctuates around the freezing and thawing points within the plant [3,4]. Owing to the cyclical generation of solid ice in plants, a large number of ice crystals will cause damage to plant cell tissues, and the generation of intracellular ice crystals causes injury or even the death of plant tissues or organs throughout the body [5,6,7,8]. When plants freeze to a lesser extent and extracellular icing occurs, the damage to plant tissue cells is not only less but also allows the plant to withstand cold, training the plant and improving its cold resistance [9,10]. This is important for the growth of forest trees in alpine regions.

In earlier studies, the freeze-thaw condition of plants was usually assessed by observing the morphology of plants under cold stress, and was mainly divided into ecological indices (half-lethal temperature and supercooling point) and morphological indices (cold-tolerance index, tissue browning, developmental index, sterility index, etc.) [11,12]. Subsequent studies have shown that physiological changes in plants under cold stress can be more accurately assessed by analyzing various biochemical metabolic indicators (enzyme activity, soluble proteins, respiration, etc.,) in plant tissues [13,14,15]. However, these methods are difficult to monitor in real-time, require expensive equipment, and have high operational requirements, making them impossible to use widely in the field. In recent years, the development and broad application of image detection techniques have provided new directions for the freezing and thawing analysis of plants under cold stress. Some scholars have used nuclear magnetic scanning imaging to observe the freezing and thawing, and some scholars have also used infrared imaging devices to observe the temperature changes of plant surfaces, and then analyze the freezing of plants [6,16,17]. The high-cost of the imaging equipment and the strict environmental conditions required for its use have led to it not being widely used in the field. To enable the online real-time monitoring of growing plants, and to measure the dynamics of cold stress experienced by overwintering plants, Sparks et al. applied a time domain reflectometry (TDR) sensor to the plant to measure the stem moisture, ice content, and xylem conductivity of pine trees. The results showed that stem ice content varied between 0% and 75% in winter [18]. Xu et al. used a TDR sensor to detect the ice content in larch and loblolly pine [19], and these results also demonstrated that the TDR sensor was able to monitor the ice content changes in plants over winter, in real-time. Sun et al. observed seasonal changes in the stem water content of apple trees over winter using a plant stem water content sensor, and developed a freeze-thaw model to calculate the changing stem ice content [20,21]. Raschi et al. proposed the use of ultrasound to detect freeze-thaw changes by analyzing the relationship between gas bursts associated with freezing in plants, and ultrasound emission [22]. Charrier et al. further traced the changes to the supercooling breakdown of xylem thin-walled cell freeze-thaw embolisms in walnut trees subjected to cold stress, by using ultrasound emission analysis [23,24]. Because of the different densities of water and ice, the cyclic conversion of liquid water to solid ice is accompanied by changes in the volume of plant stems, and since the linear variable differential transformer (LVDT) sensor can monitor changes in plant branch diameters in real-time [25], its use has been attempted for the freeze-thaw measurements of live standing trees [26,27].

The TDR, LVDT, and ultrasonic emission methods have the advantage of being nondestructive or microdestructive, and can be used in real-time in the field; however, the cost of TDR and ultrasonic emission testing equipment is prohibitive. The LVDT sensors are more suited to measuring larger diameter plants, but are not suitable for finer branches. By analyzing the impedance changes in plant branches under cold stress, we propose a method based on edge electromagnetic field induction to detect the impedance of branch tissues and thus obtain freeze-thaw information of plant branches. Additionally, a plant BVIC sensor was developed, which is not only capable of dynamic real-time monitoring, but also causes little damage to plants. The sensor is easy to install and measure on small branches, and is inexpensive (costing around USD 20), providing a technical and scientific basis for agricultural and forestry frost prevention and treatment, plant freeze-thaw physiological research, and cold resistance assessment. The main research content in this paper is as follows: (1) analyzing the relationship between the change in water-ice ratio and branch impedance during freezing; (2) developing a miniature noninvasive BVIC sensor to detect branch tissue impedance by edge-based electromagnetic field induction; (3) analyzing the measurement performance of the BVIC sensor; and (4) interpreting the freeze-thaw characteristics of plants under cold stress based on the obtained information of the branch ice content and the ice freeze-thaw rate.

## 2. Materials and Methods

### 2.1. Plant Materials

**Figure 1:**
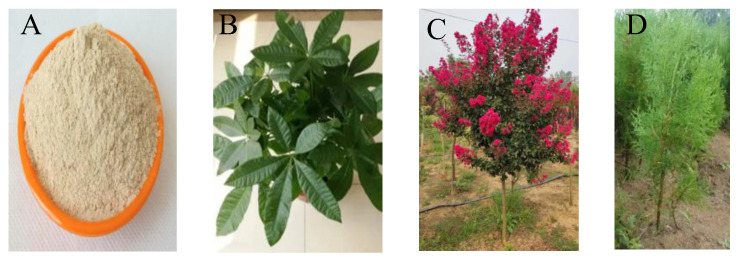
Photographs of the experimental materials used in the study. (**A**) *Populus* L. powder. (**B**) *Pachira glabra Pasq*. (**C**) *Lagerstroemia indica* L. (**D**) *Juniperus virginiana Linnaeus*.

Plant materials were divided into simulated and real. The material used for simulating plants was *Populus* L. powder (Figure 1a), which was collected from wood processing plants, with a particle size of 200 mesh. The plant branches were simulated by adding different masses of water to *Populus* L. powder and placing them in glass test tubes. The real plants used were *Pachira glabra Pasq.*, *Lagerstroemia indica* L., and *Juniperus virginiana Linnaeus*. The *Pachira glabra Pasq.* was planted in a pot in an indoor laboratory. The *Pachira glabra Pasq.* tree had a branch diameter of 3 cm, and sensor probes were installed on the stem at a height of 10 cm from the soil surface. The *Lagerstroemia indica* L. was planted on 1 July 2016. The main stem diameter was 3.5 cm at the beginning of the experiment, and the sensor probe was mounted on the branch after the first fork (branch diameter 2.6 cm) at a location 10 cm from the first fork point. The *Juniperus virginiana Linnaeus* was planted on 1 May 2017, the main stem was 2.6 cm at the beginning of the experiment, and the sensor probe was installed at a height of 120 cm from the soil surface on the main stem. Branches were cut from these trees during the nonfreeze-thaw period as isolated live wood branches and used for sensor calibration and testing.

### 2.2. Experimental Site

The indoor experimental site was located in the water-saving irrigation control laboratory of Beijing Forestry University, which has an adjustable temperature refrigerator (BD-200HEGW, Haier, Qingdao, China, temperature control range from −40 °C to 10 °C), drying oven (DHG-9030A, JINGHONG, Shanghai, China, temperature control range from 35 °C to 250 °C), high and low temperature alternating test chamber (GDJ-1500B, Beijing Chek Test Equipment Co., Ltd., Beijing, China, temperature control range from −40 to 150 °C, humidity control range from 0 to 100% RH, temperature control accuracy ±1.5 °C, humidity control accuracy ±1% RH), precision electronic scale (JE-301, HEEYII, China, accuracy 0.01 g, measuring range from 0 to 2500 g), high frequency vector network analyzer (E5070B, Agilent, California, USA, frequency range from 300 KHz to 3 GHz), conductivity meter (2265FS, Spectrum, Illinois, USA, range from 0 to 20 S/cm, accuracy 0.01 mS/cm), oscilloscope (DSOX2004A, Keysight, California, USA, bandwidth 70–200 MHz, sampling rate 2 GS/s), digital multimeter (F15B, FLUKE (China), Shanghai, China, voltage range from 0 to 1000 V, current range from 0 to 10 A), adjustable DC regulated power supply (MS305D, KANGKESI, Suzhou, China, output voltage range from 0 to 36 V, output current range from 0 to 10 A), stainless steel ruler (BTE-00722058, BANGTE, Beijing, China, measuring range from 0 to 50 cm, measuring accuracy 1 mm) and pure water (596 pure water, Wahaha, Hangzhou, China, hydrogen ion concentration 6.6, conductivity 1.08 μS/cm, total dissolved solids 0 ppm, and mineral content 0 mg/L), which were used for the experiments.

The outdoor site is located at Bajia Nursery, Haidian District, Beijing, China (116°21′14″ E, 40°0′55″ N), where *Lagerstroemia indica* L. grow in a warm, temperate, semihumid, semi-arid monsoon climate, and where the average temperature in July is 26.0 °C (hottest), whereas the average temperature in January is −5.0 °C (coldest). The other site is in Hohhot, Inner Mongolia, China (111°50′28″ E, 40°32′34″ N), where *Juniperus virginiana Linnaeus* grows, in a midtemperate, continental monsoon climate, where the average temperature in July is 24.0 °C (hottest) and the average temperature in January is −10.0 °C (coldest).

### 2.3. The Principle of Branch Volume Ice Content Measurement

**Figure 2:**
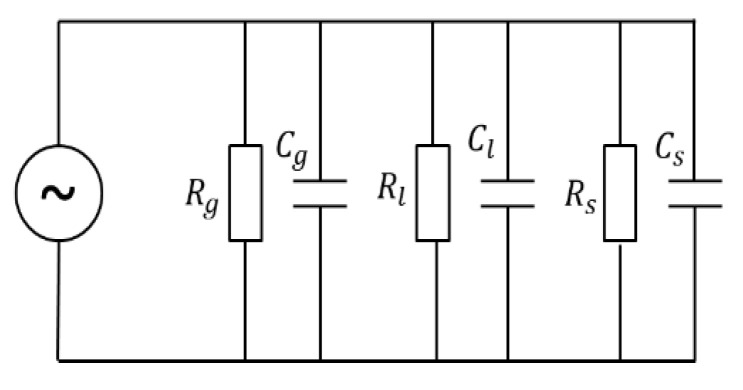
A circuit diagram of the dielectric model of the branch tissue.

The dielectric properties of plant branch tissue lie between those of polar and ionic materials, and the branch tissue is viewed as a complex porous dielectric substance consisting of a mixture of gas (g), liquid (l), and solid (s) [28,29,30], whose dielectric model can be represented by the equivalent circuit diagram, as shown in Figure 2. The total impedance of the branch tissue is:

(1) $$Z=(R_{g}//R_{l}//R_{s})+jw\left(C_{g}+C_{l}+C_{s}\right)$$

where Z is the total impedance of the branch tissue; $R_{g}$, $R_{l}$, $R_{s}$, $C_{g}$, $C_{l}$ and $C_{s}$ correspond to the resistance and capacitance of gas, liquid, and solid substances, respectively; and the expression for the relative permittivity of a capacitor is:

(2) $$\varepsilon_{r}=\frac{C}{k\varepsilon_{0}}$$

where k is determined by the geometry of the measuring electrode, $\varepsilon_{0}$ is the vacuum permittivity ($8.85\times 10^{-12}F/m$), $\varepsilon_{r}$ is the relative permittivity, and C is the capacitance. Substituting into Equation (1) yields the following Equation (3):

(3) $$Z=R+jwk\varepsilon_{0}\left(\frac{V_{g}}{V}\varepsilon_{g}+\frac{V_{l}}{V}\varepsilon_{l}+\frac{V_{s}}{V}\varepsilon_{s}\right)$$

where R is the total parallel resistance ($R_{g}//R_{l}//R_{s}$); V is the volume of the branch; and $V_{g},\;\;V_{l}\;\mathrm{and}\;V_{s}$ are the volumes of gas, liquid, and solid substances in the branch, respectively. Because the proportion of gaseous material in the branch tissue is small and the dielectric constant is much smaller than 1, its effect can be neglected, while the effect of solid material in the branch tissue on the total impedance is also small because the dielectric constant is smaller than 3 and changes very slowly. Then, Equation (3) is simplified as:

(4) $$Z=R+jwk\varepsilon_{0}\varepsilon_{l}\frac{V_{l}}{V}$$

During overwintering, along with ambient temperature changes, interconversion between liquid water and solid ice occurs within the plant branches. The dielectric constant of ice (approximately 3) is similar to that of solid matter within the branch tissue [31,32], while the dielectric constant of liquid water is approximately 81. This means when branches undergo the freeze-thaw cycle process, the liquid water $V_{l}$ dominates the imaginary part of the total impedance change of the branch tissue. Further, when a higher frequency is selected, the effect of the imaginary part in Equation (4) will be much larger than that of the real part, making Z linearly correlated with $V_{l}$. The change in liquid water before and after the freezing of the branch can be established by measuring the change in impedance of the branch tissue [20,21,33], where the amount of change in liquid water corresponds to the amount of change in solid ice, allowing the volumetric ice content of the branch to be calculated.

**Figure 3:**
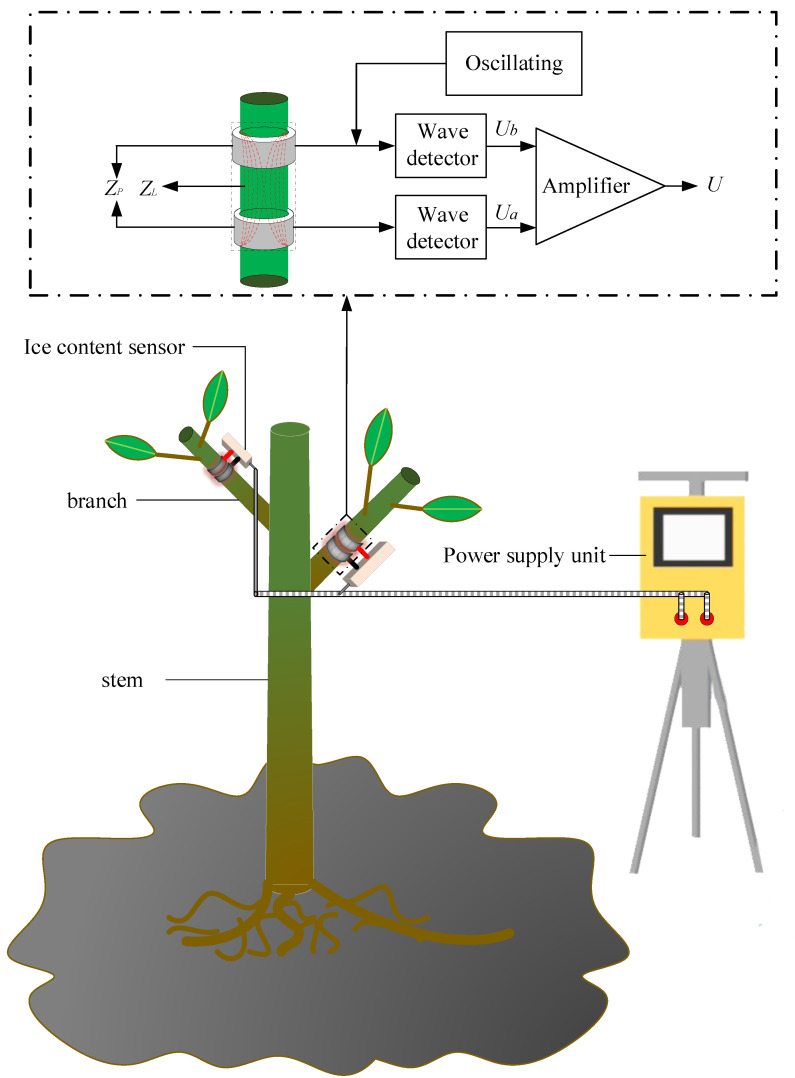
A schematic diagram of the measurement principle of the branch tissue impedance detection based on edge EMF induction.

The measurement principle for detecting the branch tissue impedance based on edge electromagnetic field induction is shown in Figure 3. When the high-frequency electromagnetic wave reaches the probe along the coaxial transmission line, the electromagnetic wave at the bimetallic ring probe changes in the transmission process, owing to the change in plant impedance between the bimetallic ring probes; the magnetic field medium changes with it, which causes the reflected electromagnetic wave to change, which in turn causes the two ends of the coaxial transmission line. The change in potential difference between the two ends of the transmission line can be found by measuring the change in voltage between ends to establish the change in the electromagnetic field at the outer edge of the probe, and thus measure the branch tissue impedance at the probe [33,34,35]. To improve the resolution of the potential difference between the two ends of the coaxial transmission line, the voltage difference between the two ends must perform signal amplification processing, and the final voltage obtained is:

(5) $$U=\beta_{0}\left(U_{a}-U_{b}\;\right)=2\times \beta_{0}\times A\times (Z_{P}-Z_{L})/(Z_{P}+Z_{L})$$

where U is the potential difference between the two ends of the coaxial transmission line; $\beta_{0}$ is the amplification; A is the excitation signal source amplitude; $Z_{P}$ is the impedance at the probe at the measurement electrode; $Z_{L}$ is the coaxial transmission line impedance (50 Ω); $\beta_{0}$, A, $Z_{L}$ are fixed values; U is only related to $Z_{P}$; and the size of $Z_{P}$ is determined by the branch impedance and electrode impedance. Once the electrode material and size are determined, since the impedance of the electrode itself is also a fixed value, U is related only to the branch impedance. By measuring the change in U, the change in the branch impedance during freezing can be measured and the change in the branch ice content determined.

Because the dielectric constant of ice (approximately 3) is similar to that of the solid material in the branch tissue, while the dielectric constant of liquid water is approximately 81, the impedance of the branch during freezing and melting is principally affected by the percentage of liquid water in the branch. This implies the percentage of liquid water is related to the circuit output voltage U. In order to facilitate field application, it is necessary to calibrate the different measurement types in advance, and establish the relationship between the branch volumetric water content and voltage U, as in Equation (6). During overwintering, by measuring the volumetric water content in the branch before freezing, and the volumetric water content during the freezing process, the volumetric ice content in the branch at any moment can be calculated by substituting the drop in the volumetric water content in Equation (7):

(6) θ=k×U+b

(7) $$\delta =\left(\theta_{0}-\theta_{x}\right)\times \frac{\rho_{ice}}{\rho_{water}}\;$$

where θ is the volumetric water content within the branch; k and b are calibration coefficients; δ is the volumetric ice content within the branch; $\theta_{0}$ is the volumetric water content of the branch above freezing point; $\theta_{x}$ is the volumetric water content of the branch during freezing; and $\rho_{ice}$ and $\rho_{water}$ are the densities of ice and water, respectively.

Excessively rapid freezing in the plant during freeze-thaw will lead to massive tissue cell death, which in turn will cause irreversible damage to the plant; therefore, the ice freeze-thaw rate of the branches is an important indicator of the freeze-thaw characteristics of plants. The formula is:

(8) $$\sigma_{tx}=\frac{\left|\delta_{tx^{\prime }}-\delta_{tx}\right|}{\left|tx^{\prime }-tx\right|}$$

where $\sigma_{tx}$ is the ice freeze-thaw rate of the branch at time tx; and $\delta_{tx^{\prime }}$ is the volume ice content of the branch corresponding to $tx^{\prime }$ at an adjacent time after tx.

### 2.4. Impedance Variation at Different Frequencies

The selection of the appropriate measurement frequency plays an important role in improving measurement accuracy. A related study by Hilhost and Driksen [36] demonstrated that the effects of the variability between plant species can be significantly reduced between 100 MHz and 500 MHz. From existing crystal chip frequencies that are readily available in the market, eight frequencies of 50 MHz, 80 MHz, 100 MHz, 150 MHz, 200 MHz, 250 MHz, 300 MHz, and 400 MHz, were selected to simulate the branches. Live wood branches (*Lagerstroemia indica* L. branches) configured with different volumetric moisture content simulated the branches. The different volumetric ice contents during freezing were measured by the vector network analyzer E5070B to determine the excitation frequency of the impedance measurement circuit, by measuring the changes in the real and imaginary parts of the branch impedance. These corresponded to each moisture gradient under the different signal frequency excitation of the designed probe structure. The simulated branches were made by mixing *Populus* L. powder uniformly with different amounts of water, and then placing a portion of the sample into a 20 mm diameter test tube and wrapping it with plastic wrap to prevent water loss. The volumetric water content of the branches was 0%, 1.62%, 3.64%, 5.52%, 7.85%, 13.93%, 19.14%, 25.20%, 40.10%, 50.07%, and 74.99%, for the 12 gradients of sample, where 0% is the air in the test tube and 74.99% is the volumetric water content of the sample at saturation. Live wood branches were pruned from healthy *Lagerstroemia indica* L. plants, were soaked in water for 12 h, and then tested experimentally. After one impedance measurement, the samples were put into the drying chamber (DHG-9030 45 °C, 6 h), and then removed to enable the branches to return to room temperature. The mass of the samples after drying, and the impedance of the samples at different frequencies, were measured before they were placed in the drying chamber. This process was repeated until the branches of *Lagerstroemia indica* L. were thoroughly dried, and the change in the mass of water in the samples was obtained by weighing and dividing by the density of water to obtain the volume of water in the samples. The volumetric water content of the samples was further calculated to obtain a total of 20 variants of volumetric water content in isolated live wood branches, which were 0%, 0.38%, 1.28%, 1.33%, 2.99%, 4.30%, 5.66%, 10.67%, 14.66%, 21.84%, 28.26%, 32.56%, 36.33%, 40.45%, 43.58%, 46.22%, 49.03%, 52.69%, 56.01%, and 58.36%.

### 2.5. Impedance Changes under Cold Stress

The *Populus* L. powder was selected and a sample of mock branches with a volumetric moisture content of 45% was configured. The impedance measurement probe was mounted on the simulated branches and these were placed whole into the adjustable temperature refrigerator BD-200HEGW. The temperature was set to drop from 20 °C to −16 °C, and then rise to 20 °C when it had reached −16 °C. The process of cold stress was simulated by this cooling and warming, with the temperature adjustment rate set to 3 °C every 10 min. Impedance change in the samples during the freezing and thawing was measured with a vector network analyzer E5070B (measurement frequency 100 MHz). Fresh branches of *Lagerstroemia indica* L. were pruned and wrapped with cling film immediately after cutting to prevent moisture loss. After the begonia branches were collected, the measurement probe was mounted on them and they were placed in the low-temperature refrigerator (BD-200HEGW). The temperature was set to drop from 20 °C to −20 °C, and then rise to 20 °C when it reached −20 °C. The temperature adjustment rate was set so that every 10 min the temperature changed by 2 °C, and the impedance change of the samples during this freeze-thaw process was measured with a vector network analyzer E5070B (measurement frequency of 100 MHz).

### 2.6. Impedance Measurement Circuit Performance Analysis

**Figure 4:**
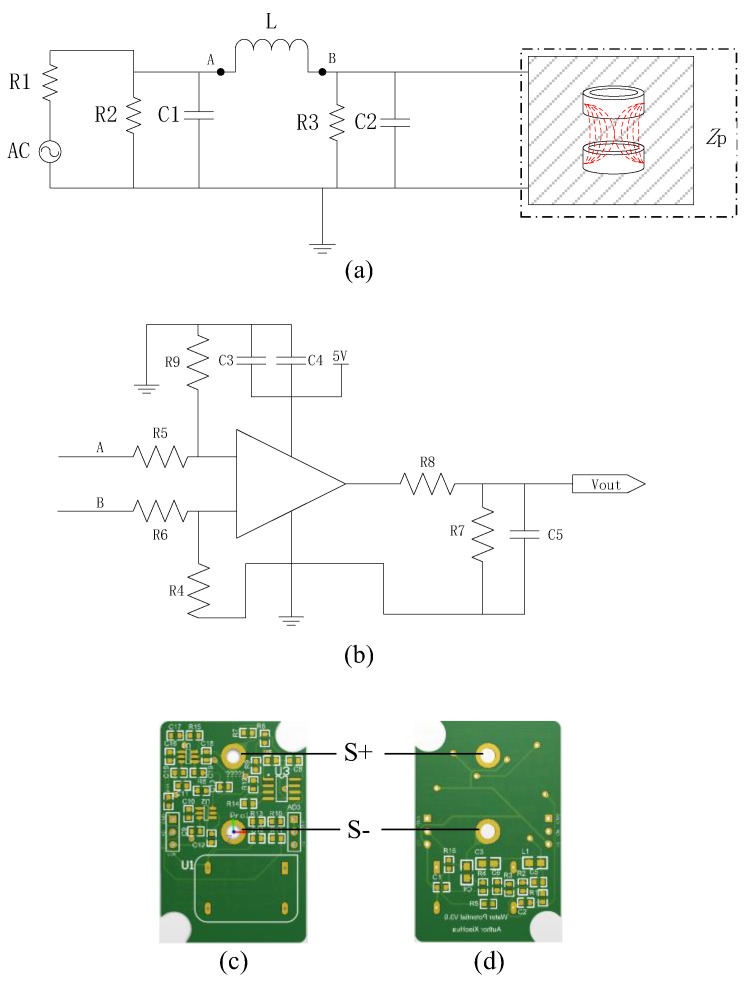
The impedance measurement circuit: (**a**) a schematic diagram of the impedance sensing circuit; (**b**) a schematic diagram of the signal amplification circuit; (**c**) the front of the PCB; (**d**) the back of the PCB. S+ and S− are the link fixing holes for the positive and negative terminals of the probe, respectively.

The impedance measurement circuit consists of a detection circuit (impedance sensing circuit and signal amplification circuit) and a measurement probe. A schematic diagram of the impedance sensing circuit is shown in Figure 4a. A schematic diagram of the signal amplification circuit is shown in Figure 4b, and the output signal is an analog voltage signal. The final design of the detection circuit printed circuit board (PCB) is shown in Figure 4c,d. The PCB board size was 28.5 mm × 44 mm.

After completing the impedance measurement circuit design, performance tests were conducted to ensure the reliability of the measurement results. Static characteristics indicate the relationship between the sensor output and input when the input is constant, or largely unchanged. For application in the scenario of the branch ice content measurement, the static characteristic test focuses on the analysis of the measurement sensitivity range, measurement range, resolution, measurement accuracy, and power consumption. In order to visually characterize the sensor radial sensitivity range changes, this paper uses the energy index $K_{w}$ to indicate the energy distribution on the radial range. The energy index calculation formula is as follows:

(9) $$K_{w}=(U_{c}-U_{x})/(U_{c}-U_{0})\times 100\%$$

where $U_{x}$ is the voltage output from the sensor when different diameter branches are placed in the center of the test tube (different diameter water columns are used to simulate different diameter branches); $U_{0}$ is the sensor output voltage when the test tube is in air; and $U_{c}$ is the sensor output voltage when the test tube is in water.

Dynamic characteristics indicate the response characteristics of the sensor to input quantity changes with time. By measuring the probe empty value in the air to be its output stability, then quickly placing the probe into a beaker containing 500 mL of water (a process which can be seen as a step input signal), and by using an oscilloscope to observe the sensor output signal changes, we can establish the dynamic characteristics of the impedance measurement circuit output curve.

The actual use environment is often complex and harsh, and the plant’s own electrical conductivity may also cause changes in plant tissue characteristics, as shown in Table 1. The GDJ-1000B high- and low-temperature alternating humidity and heat test chamber was used to set four different temperature environments. Different humidity levels were set at each temperature, in which the impedance measurement circuitry was used, and its output recorded every 5 min under each temperature and humidity environment. The voltage results were measured every 5 min in each temperature and humidity environment, with a total of 200 measurements taken to analyze the response of the impedance measurement circuit to environmental temperature and humidity changes. Aqueous sodium chloride solutions of various concentrations were also configured in test tubes and beakers to simulate the different conductivities of the branch tissue solutions. The conductivity of the configured aqueous sodium chloride solutions ranged from 0.53 S/cm to 16 mS/cm, measured using a 2265FS conductivity meter, and the response of the impedance measurement circuit to changes in the branch conductivity was analyzed. To better observe the change in conductivity, let Δ**φ_*n*_** denotes the value of the change in sensor output, with the equation being:

(10) $$\Delta {𝛗}_{n}=U_{n}{}^{\prime }-U_{0}{}^{\prime }$$

where $U_{n}{}^{\prime }$ is the measured value of the impedance measurement circuit at the nth conductivity gradient; and $U_{0}{}^{\prime }$ the measured value of the impedance measurement circuit at the first conductivity gradient.

### 2.7. The BVIC Sensor Hardware System

**Figure 5:**
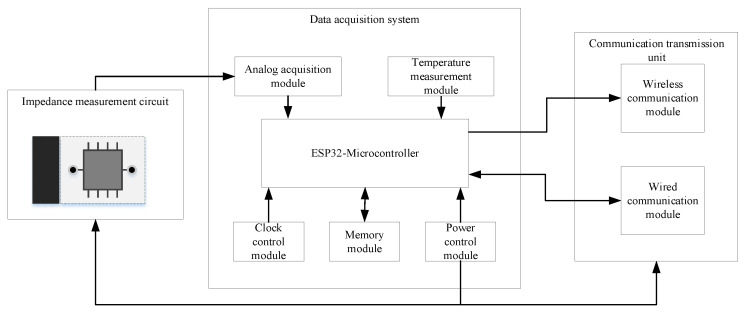
A schematic diagram of the overall hardware circuit system.

The BVIC sensor hardware system consists of an overall hardware circuit system and a mechanical structure. The overall hardware circuit of the BVIC sensor is shown in Figure 5. The impedance measurement circuit is used for measuring the branch impedance and outputting analog voltage values after signal conversion. The data acquisition system consists of a microcontroller (ESP32-S3-WROOM-1, Espressif Systems, Shanghai, China), an analog acquisition module (AD7705, Analog Devices Inc., Wilmington, MA, USA), a clock control module (RX-8025T, Epson Toyocom, Nagano, Japan), a memory module (AT24C256, TELESKY, Shenzhen, China), a power control module (K7805-1000R3, Dexu electronics, Shenzhen, China), and a temperature measurement module (DS18B20, Risym, Shenzhen, China). The microcontroller is used to control data acquisition, calibration, and calculation. The analog acquisition module is used to convert the voltage value output from the impedance measurement circuit into a digital quantity for calculation and processing by the microcontroller. The clock module provides a reference for controlling the data acquisition time. The memory module is used to store calibration coefficients, historical data, and command protocols. The power supply module is used to step down the external input voltage to supply power to the overall hardware of the sensor. The power supply module is used to step down the external input voltage to power the overall hardware circuit system of the sensor. Finally, the ambient temperature module is used to determine whether the branch is in a frozen state by measuring the ambient temperature. The communication transmission unit consists of a wireless communication module (WH-LTE-7S1, USR-IOT, Jinan, China) and a wired communication module (HT8487EARZ, HTCSEMI, Shenzhen, China); the wireless communication module is for uploading data to the cloud database, while the wired communication module is used for communication with other data collection devices.

**Figure 6:**
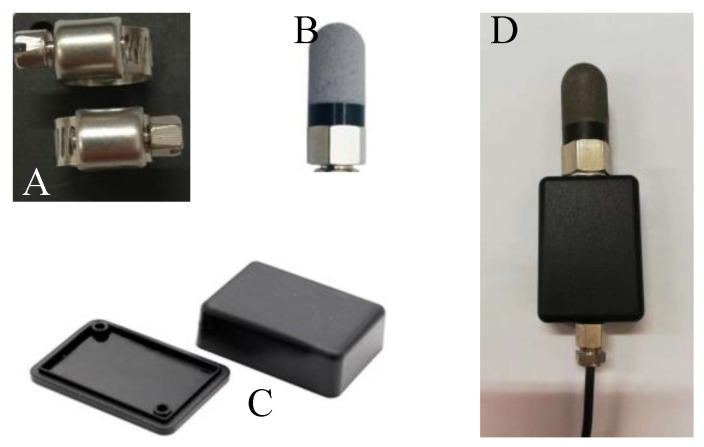
**The** BVIC sensor mechanical structure. (**A**) A bimetallic ring gauging probe. (**B**) The waterproof protective cover for the temperature probe. (**C**) Sensor housing. (**D**) The assembled BVIC sensor.

The BVIC sensor mechanical structure contains a bimetallic ring measuring probe, a temperature probe, a waterproof protective cover and sensor housing, and a ring measuring probe, as shown in Figure 6a. It is made of ASTM 304 stainless steel. The width is 12.6 mm and the thickness is 0.65 mm. Through the adjustment knob on the electrode, the diameter of the ring electrode is adjustable in a range from 10 to 40 mm, the corresponding surface coverage areas ranged from 791.28 mm^2^ to 3165.12 mm^2^ (the surface coverage area is as in Formula (11)), so that it can adapt to different diameters of the branches. The waterproof protective cover of the temperature probe, with a length of 45 mm and an outer diameter of 16.7 mm, is shown in Figure 6b. The BVIC sensor housing is made of 3D printing resin material, with a printing tolerance of 0.2% and outer dimensions (length × width × height) of 51 mm × 36 mm × 20 mm, as shown in Figure 6c. The assembled BVIC sensor is shown in Figure 6d.

(11) $$S=2\pi R_{branch}h$$

where S is the surface coverage area; $R_{branch}$ is the diameter of the ring electrode; and h is the width of ring electrode.

### 2.8. BVIC Sensor Calibration

The branches were selected from healthy *Pachira glabra Pasq.*, *Lagerstroemia indica* L., and *Juniperus virginiana Linnaeus* plants growing in good conditions, and the volume of the branches was measured by the overflow method. The BVIC sensor was mounted on the branches and they were placed in a drying oven (DHG-9030A) for drying (45 °C), after which the branches were removed and weighed every 6 h to record the voltage value output from the impedance measurement circuit in the BVIC sensor circuit, until the branches had completely dried. The volumetric water content (true value) obtained by the voltage value, and the drying method, was linearly fitted to obtain the calibration coefficients k and b in Equation (6), and was written into the memory of the BVIC sensor. For measuring the branch ice content over winter, by obtaining the output voltage value of the branch impedance measurement circuit in real-time, then the volumetric water content of the branch before the freezing point, and the volumetric water content during the freezing process, can be calculated. The model of Equations (7) and (8) is embedded in the sensor memory through the program code. The BVIC sensor measures in real-time whether the ambient temperature reaches the freezing point during operation, and then calculates the volumetric ice content and ice freeze-thaw rate of the branch at any moment. Calibration improves the consistency of the sensor’s output, allowing measurements to be displayed as volumetric ice content and ice freeze-thaw rate; it also helps to check to exclude defective products from the sensor hardware manufacturing process.

### 2.9. The Ice Content of Plant Branches under Cold Stress

In the indoor simulated cold stress experiment, the BVIC sensor designed in this study was mounted on living *Pachira glabra Pasq.* The whole *Pachira glabra Pasq.* was placed in a low-temperature refrigerator (BD-200HEGW), and the refrigerator temperature was set to −30 °C. After 10 h, the power of the refrigerator was turned off and the refrigerator was opened, allowing the temperature to rise naturally. The ice content and ice freeze-thaw rate of *Pachira glabra Pasq.* were measured every 1 min to observe the changes in the branch volume ice content and the ice freeze-thaw rate when *Pachira glabra Pasq.* cells were exposed to cold stress.

In the field cold stress experiment, the BVIC sensors were mounted on *Lagerstroemia indica* L. and *Juniperus virginiana Linnaeus* from 25 October, 2018, to 10 March, 2019. Data were recorded once every 10 min to capture changes in the branch volume ice content and the ice freeze-thaw rate in *Lagerstroemia indica* L. and *Juniperus virginiana Linnaeus* during the overwintering period, when they experienced cold stress.

## 3. Results

### 3.1. Variation in the Branch Impedance at Different Frequencies

**Figure 7:**
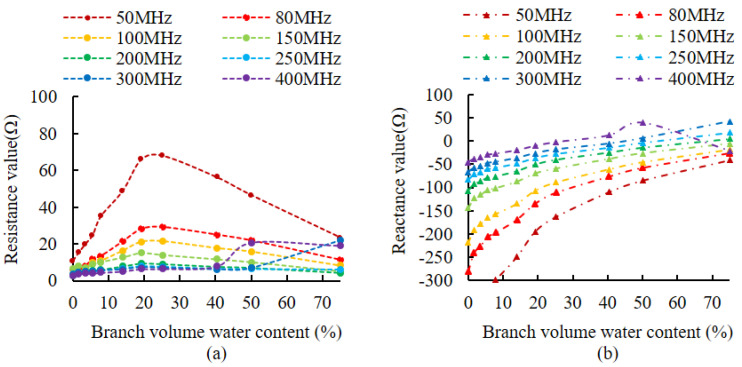
The variation of the impedance of the simulated branches. (**a**) The variation of the resistance component. (**b**) The variation of reactance components.

**Figure 8:**
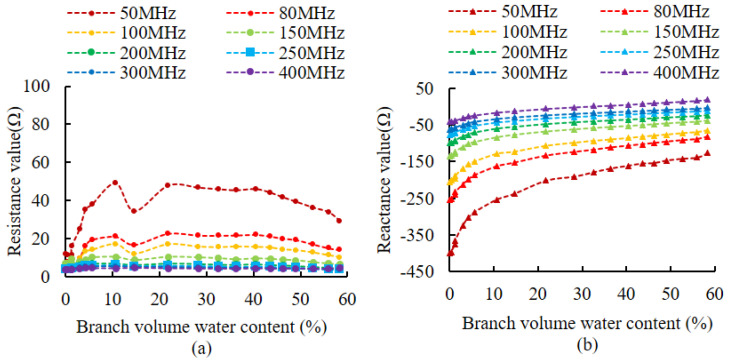
The variation of the impedance of the live wood branches. (**a**) The variation of the resistance component. (**b**) The variation of reactance components.

Impedance values were measured for simulated branches and live wood branches at different volumetric water contents, using different signal frequencies as excitation. Figure 7 shows the impedance variation curve of the simulated branches, and Figure 8 shows the impedance variation curve of the live wood branches. Figure 7a shows that in the range from 100 MHz to 250 MHz, the resistance component fluctuates in a range from 0 to 20 Ω, and in the other frequency ranges, the resistance component fluctuates in a range of more than 0 to 20 Ω. Figure 7b shows that in the range from 50 MHz to 200 MHz, all values of the reactance component are less than 0. Figure 8a shows that the live wood branch resistance components fluctuate in a range from 80 MHz to 400 MHz, all in a range from 0 to 20 Ω. Figure 8b shows that the reactance components of the live wood branch were less than 0 in the range from 50 MHz to 250 MHz.

### 3.2. Changes in the Impedance of Branches under Cold Stress

**Figure 9:**
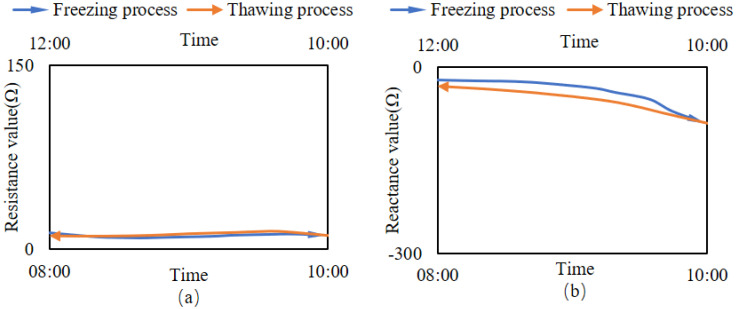
Changes in the impedance of the simulated branches during freeze-thaw cycles. (**a**) The variation of the resistance component. (**b**) The variation of reactance components.

**Figure 10:**
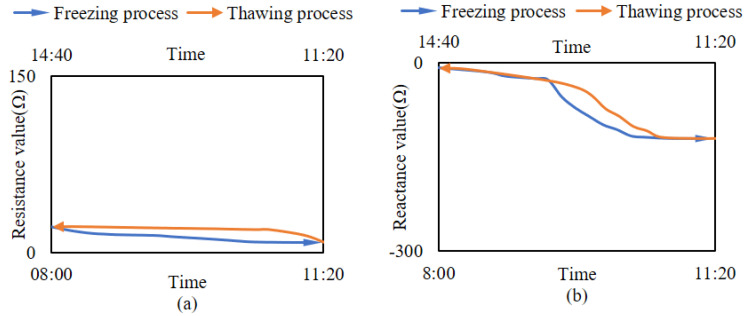
Changes in the impedance of the live wood branches during freeze-thaw cycles. (**a**) The variation of the resistance component. (**b**) The variation of reactance components.

Simulating the environment where the branches encounter cold stress, the impedance changes of the simulated branches and live wood branches during the freeze-thaw cycle are shown in Figure 9 and Figure 10. Figure 8 illustrates that the resistance component of the simulated branches was essentially constant during freezing and thawing, whereas the reactance component decreased gradually during freezing and increased gradually during thawing. Figure 10 illustrates that during the freeze-thaw cycle of live wood branches, the resistance component gradually decreases during the freezing process, and gradually increases during the thawing process. However, the overall change in the resistance component during the freeze-thaw cycle is very small; the reactance component decreases slowly during the freezing process and then decreases rapidly, and finally stabilizes gradually, while the change in the reactance component during the thawing process is the opposite.

### 3.3. The Static Characteristic Performance and Dynamic Characteristic Performance of Impedance Measurement Circuits

**Figure 11:**
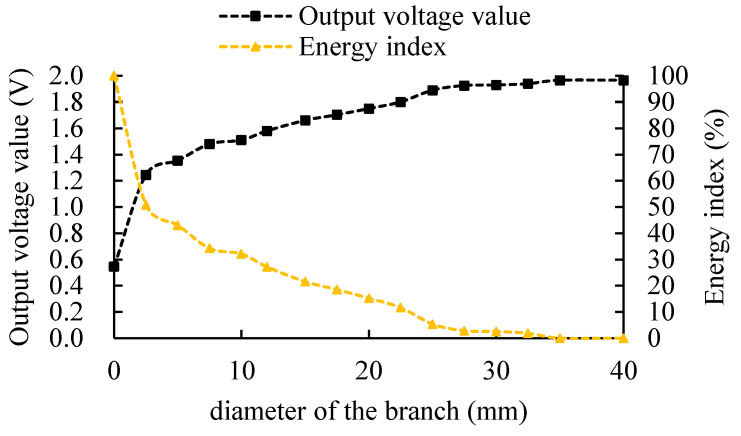
The output voltage and energy index variation of the impedance measurement circuit.

The impedance measurement circuit output voltage and the energy index, and their relationship to the diameter of the branch, are shown in Figure 11. The results suggest that with the increasing diameter of the branch, the BVIC sensor output voltage value first gradually increases and then remains constant, the energy index gradually decreases, and when the diameter of the branch is 35 mm the energy index becomes 0%. This indicates that when the probe is measuring a sample diameter of more than 35 mm, the sensor will not be able to effectively detect the branch changes, meaning the sensor radial sensitivity range is 35 mm. In actual use, the longitudinal length of live-standing branches is much larger than the radial diameter. Since the sensor is mounted on the branch, and its sensed longitudinal range does not change, the longitudinal sensitivity range can be ignored.

The measurement result of the BVIC sensor in the unloaded state is taken as 0%, and the ideal state of all ice in the branch is regarded as 100% ice content. When the BVIC sensor is installed in a test tube of water and put into the freezer to freeze, the output voltage of the branch impedance circuit first decreases gradually with the increase of ice in the test tube, and continues until the water in the test tube is completely frozen, from where it does not change. This means the BVIC sensor can effectively record the entire change process during water freezing; that is, the BVIC senses the volume ice content over the range from 0% to 100%. During the complete transformation of water from liquid to solid in the test tube, the difference in the voltage change was 1.574 V. It was calculated that each 1 V change in voltage value represents a 63.53% change in ice content, and the sampling resolution of the analog-to-digital conversion module was 0.0008 V. Therefore, the resolution of the BVIC sensor was 0.05% (63.53%/V × 0.0008 V). It also shows that the BVIC sensor can respond sensitively to changes in the sample volume ice content above 0.05%. Three different volumes of water content of the branches (0%, 3.55%, and 100%) were configured to simulate the freezing process of different ice contents of the branches. The BVIC sensor was installed on the branches, with the sensor output measured at 2 s intervals. A total of 200 measurements were taken and the standard deviation of the measurement results was calculated. The fluctuation range of the measurement error of the BVIC sensor during the measurement of the three samples was recorded, and the measurement accuracy calculated. Measurement accuracy was defined as the ratio of the maximum error in the measurement range to the whole measurement range, which was calculated, and the maximum value was taken to establish a measurement accuracy of 3.52%. Measurement accuracy was usually expressed as a positive or negative range, meaning the measurement accuracy of the BVIC sensor was ±1.76%. Since the BVIC sensor supply voltage is 5 V and operating current is 50 mA, the power consumption of the BVIC sensor is 0.25 W (the product of current and voltage per unit time being power consumption).

**Figure 12:**
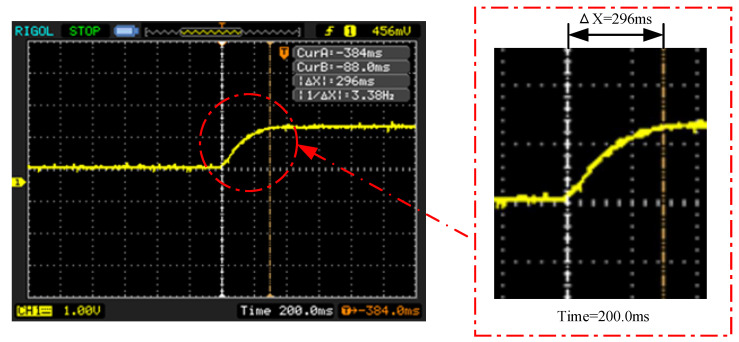
The dynamic characteristic curve of the output of the impedance measurement circuit.

The dynamic characteristic curve of the output of the impedance measurement circuit was recorded using an oscilloscope, and the results are shown in Figure 12. The ∆X was 296 ms, indicating that the dynamic response time of the impedance measurement circuit was 0.296 s.

### 3.4. The Response of Impedance Measurement Circuits to Ambient Temperature and Humidity Changes

**Figure 13:**
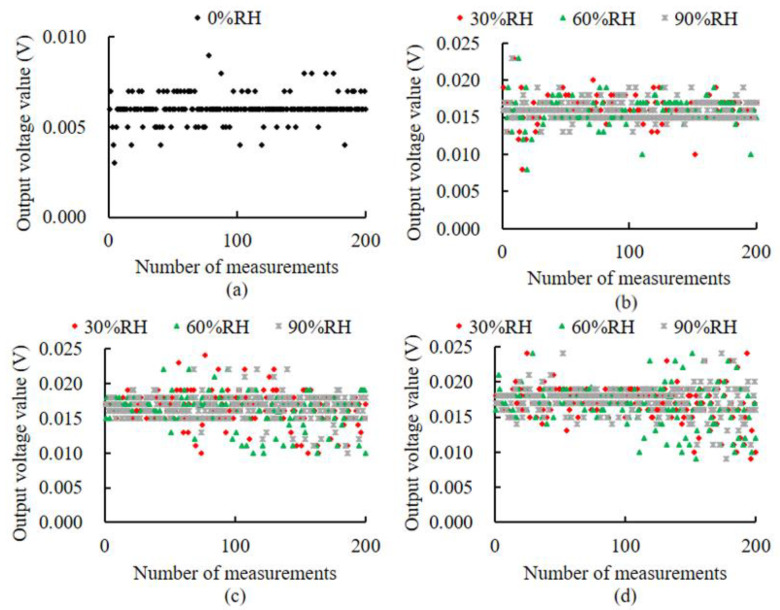
The response of the impedance measurement circuit to ambient temperature and humidity. (**a**) −40 °C. (**b**) 5 °C. (**c**) 35 °C. (**d**) 65 °C.

The output voltage variations under different temperatures and humidities are shown in Figure 13. As can be seen in Figure 13a, the output voltage fluctuates from 0 V to 0.01 V at different temperatures when the environment is 0% RH. The output voltage fluctuates in a range from 0.005 V to 0.025 V at different temperature and humidity, as shown in Figure 13b–d. From the resolution of the impedance measurement circuit, it is known that each 1 V change in the output value represents a 63.53% change in volume water content, meaning voltage fluctuations in a range from 0.005 V to 0.025 V correspond to a volume water content fluctuation range from 0.32% to 1.59%. The maximum allowable error caused by fluctuations in the output voltage due to ambient temperature and humidity is ±0.635%.

### 3.5. The Response of Impedance Measurement Circuits to Changes in Branch Conductivity

**Figure 14:**
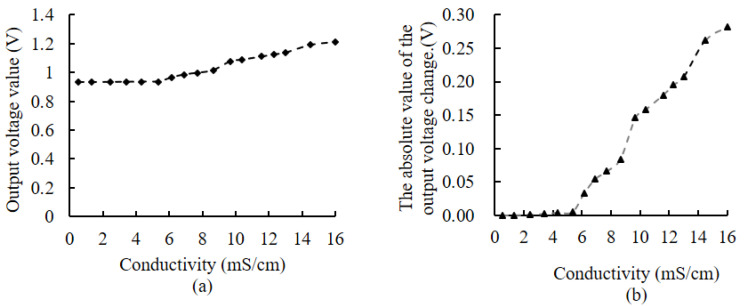
The response of the impedance measurement circuit to changes in the branch conductivity. (**a**) The variation of output voltage with conductivity. (**b**) The variation of the absolute value of the output voltage change (Δ**φ_*n*_**) with conductivity.

Aqueous solutions of sodium chloride at various gradient concentrations were configured in test tubes to simulate the branch tissue solutions with different conductivities. The variation curves of the output voltage of the impedance measurement circuit and its absolute change value Δ**φ_*n*_** are provided in Figure 14. This shows that when the conductivity is less than 6 mS/cm, the output result of the impedance measurement circuit does not change with the increase in conductivity, indicating that the effect of conductivity on the impedance measurement circuit is small at low conductivity.

### 3.6. The Calibration of the BVIC Sensor

Using the previously described method [18,37], the results of fitting the voltage output from the impedance measurement circuit of the BVIC sensor circuit to the volumetric water content, are shown in Table 2. This indicates a clear linear relationship between voltage values and the volumetric water content of the branches. The one-time linear fit coefficient of determination reached 0.98 or more, which indicates the BVIC sensor can effectively measure the variation in the volumetric water content of the branches. In combination with the ice content calculation model, the *k* and *b* values of the fitted equations and the measurement model were embedded in the microprocessor of the sensor, and the branch volume ice content was calculated in real-time from the branch volume water content.

### 3.7. The Variation in Ice Content of the Branches of Pachira glabra Pasq. under Indoor Test Conditions

**Figure 15:**
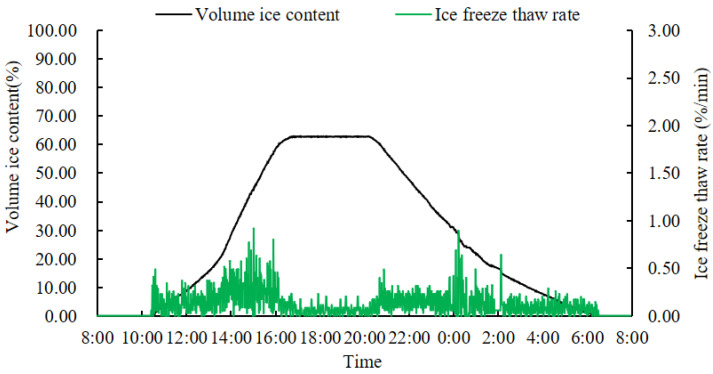
The variation of the branch volume ice content versus the ice freeze-thaw rate in *Pachira glabra Pasq.*

Changes in the volumetric ice content of the branches of *Pachira glabra Pasq*. in indoor simulated cold stress experiments are shown in Figure 15. This shows the ice freeze-thaw rate fluctuated greatly during the increasing volume ice content phase; after complete freezing the volume ice content of *Pachira glabra Pasq.* was effectively stable, while the fluctuation in ice freeze-thaw rate was very small. After the thawing stage, the volume ice content decreased rapidly, and the fluctuation in ice freeze-thaw rate increased.

### 3.8. Changes in the Branch Volume Ice Content of Lagerstroemia indica L. and Juniperus virginiana Linnaeus during the Overwintering Period

**Figure 16:**
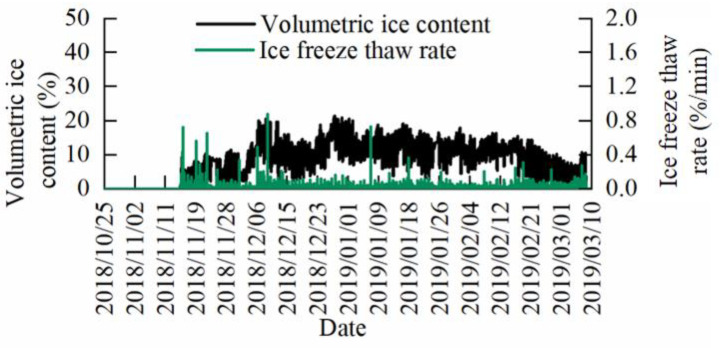
The variation of volumetric ice content and the ice freeze-thaw rate of the branches of *Lagerstroemia indica* L.

**Figure 17:**
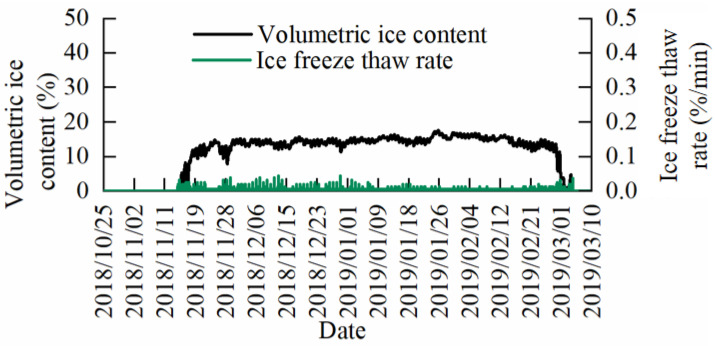
The variation of volumetric ice content and the ice freeze-thaw rate of the branches of *Juniperus virginiana Linnaeus*.

The changes in the branch volume ice content of *Lagerstroemia indica* L. during overwintering cold stress are shown in Figure 16, and the changes in the branch volume ice content of *Juniperus virginiana Linnaeus* during overwintering cold stress are shown in Figure 17. Before entering the overwintering period, the temperature was high and the plants were not subjected to cold stress, meaning the volume of ice in the branches was zero. With winter, the temperature dropped and the liquid water in the plants was transformed into solid ice by cold, causing the volume of ice in the branches to gradually increase. By late winter, the temperature is lower and the volume of ice in the branches further increases and fluctuates within a certain range. Meanwhile, in the spring, when the temperature rises, the solid ice in the plants begins to melt, and the volume of ice in the branches gradually decreases until the winter period is completely over. At this time, the solid ice in the plants disappears, and the volume of ice in the branches becomes 0. Fluctuations in the branch volume ice content during the overwintering period of *Lagerstroemia indica* L. and *Juniperus virginiana Linnaeus* were significantly different, and it was observed that *Juniperus virginiana Linnaeus* had little fluctuation, while *Lagerstroemia indica* L. showed more fluctuation. Comparing the ice freeze-thaw rate variation of *Lagerstroemia indica* L. and *Juniperus virginiana Linnaeus* in Figure 16 and Figure 17, the results of the statistical analysis of ice freeze-thaw rate volatility (Table 3), it can be seen that the ice freeze-thaw rate of *Juniperus virginiana Linnaeus* fluctuated very little, with a standard deviation of only 0.00405%/min. Meanwhile, the ice freeze-thaw rate of *Lagerstroemia indica* L. trees fluctuated more, with a standard deviation of 0.02256%/min.

## 4. Discussion

### 4.1. The Effect of Different Frequencies on Branch Impedance Measurements

According to Equation (4), in order to ensure good measurement results, the resistance component should be a fixed value and only the reactance component is linearly related to the impedance value. However, in practice, the variation of water in the branch will also lead to the variation of the resistance component. We would like, therefore, to try to find a suitable excitation frequency at which the resistance component is small and varies very little (it can be approximated as a fixed value and its effect can be neglected), while the reactance component varies as linearly as possible. The results in Figure 7a and Figure 8a show that the resistance component fluctuates less than 20 Ω when the impedance measurement excitation is in a range from 100 MHz to 250 MHz, and when the resistance value is small and changes little, so the optimal frequency for the resistance component is 100 MHz to 250 MHz. Figure 7a and 8a also show that as the volume water content increases, the higher the frequency, the smaller the resistance component and the fluctuations are smaller. This proves that the higher the frequency of the excitation source, the greater the output energy, the greater the intensity of the electromagnetic field between the measurement probes and the easier to penetrate the dielectric material. However, the higher the frequency of the excitation source, the greater the power consumption of the instrumentation, so the choice of frequency should be considered in conjunction with the specific application object.

For the reactance component, when its value is negative, this indicates the object under test is capacitive. When it is positive it indicates that the object under test is inductive, implying the reactance component in the measurement should be negative. Comparing Figure 7b and Figure 8b, only when the excitation is in a range from 50 MHz to 200 MHz are all values of the reactance component less than 0. This indicates that there is no change from capacitive to inductive in the measured object in the range from 50 MHz to 200 MHz. At the same time, the reactance component increases with the increase in volume water content, so the optimal frequency of the reactance component is from 50 MHz to 200 MHz. The reactance component changes as much as possible to improve the resolution of the measurements [21,36]. Figure 7b and Figure 8b show that as the volume of water content increases, the smaller the frequency, the greater the change in the reactance component, so for reactance component consideration, we should preferably choose a smaller frequency. Considering the experimental results corresponding to the simulated branches and live wood branches, since it is necessary to make both the resistance component and reactance component meet the requirements, the common part of the two frequency ranges (from 100 MHz to 200 MHz) is selected as a feasible frequency range. This supports the conclusion of Hilhost and Driksen’s study [36]. Considering the complexity of the circuit implementation and the price and cost of the crystal chip, 100 MHz was finally chosen as the circuit frequency.

### 4.2. Changes in the Impedance of Branches

Figure 9 and Figure 10 show that the electrical resistance component of the branch is constant during freeze-thaw, while the electrical resistance component varies periodically, indicating that the change in the internal water-ice ratio when the branch is subjected to cold stress mainly affects the imaginary part of the impedance value. This result demonstrates exactly the change in the total impedance value of the imaginary part of the plant tissue dominated by the high frequency excitation source in Equation (4). This indicates that the change in liquid water before and after freezing can be detected from the change in the impedance of the branch tissue [21,33], and calculating the amount of change to obtain the branch volume ice content is feasible. In Figure 9 and Figure 10, the gradual decrease of the electrical resistance during freezing indicates that the liquid water within the branches becomes ice under cold stress. The increase in the ice versus the decrease in the water causes a decrease in the dielectric constant of the total ice-water mixture, proving the existence of ice crystals in plants under cold stress [5,6,7].

### 4.3. The Performance Characteristics of BVIC Sensors

The static characteristics test indicated that the sensitivity range of the impedance measurement circuit is 0–35 mm, the measurement range is 0–100%, the resolution is 0.05%, the measurement accuracy is ±1.76%, and the power consumption is 0.25 W. The dynamic characteristics test obtained a response time of 0.296 s. Compared to similar sensors, the BVIC sensor designed in this study meets practical use requirements [18,21,37]. Figure 13 illustrates the error caused by output voltage fluctuation, due to ambient temperature and humidity, is only ±0.635%, which indicates that temperature and humidity have little effect on the impedance measurement, suggesting the reliability of results. Figure 14 shows that when conductivity is greater than 6 mS/cm, the BVIC sensor output gradually increases with the increase of conductivity, indicating the conductivity will have a significant impact on the measurement results of the BVIC sensor. When used to measure the plant branches with high conductivity, corrections of the BVIC sensor are required. The subjects of this paper are mainly woody plants in nonsaline areas, whose branch conductivity are significantly less than 6 mS/cm [38], therefore, the effect of conductivity on the BVIC sensor can be disregarded.

### 4.4. The Significance of the Ice Content and the Ice Freeze-Thaw Rate Measurements for Overwintering Woody Plants

The volumetric ice content and the branch freezing and thawing rates of *Lagerstroemia indica* L. and *Juniperus virginiana Linnaeus* over winter (Figure 16 and Figure 17) show the volumetric ice content in stems increases continuously from autumn to winter, as the plants enter dormancy and vitality decreases. Further, it decreases from winter to spring, as the plants begin to recover and vitality increases, following the general pattern of plant growth [39]. Over winter, the volumetric ice content of the stem fluctuates, with ice melting in the stem. A decrease in volumetric ice content as temperatures rise during the day, and water freezes in the stem with an increase in ice content as temperatures decrease at night, confirms previous reports of periodic fluctuations in ice content in the branches [18,21,32,40]. Comparing the changes in the branch volume ice content and the ice freeze-thaw rate of *Lagerstroemia indica* L. (a deciduous broadleaf tree) and *Juniperus virginiana Linnaeus* (an evergreen coniferous tree), shows the branch volume ice content of *Juniperus virginiana Linnaeus* is less volatile during the overwintering period, and its corresponding ice freeze-thaw rate is small. The standard deviation of the ice freeze-thaw rate is only 0.00405 (Table 3), indicating that over winter evergreen coniferous trees are able to regulate themselves more effectively than deciduous broadleaf trees. Further, they are able to regulate their water-ice content more effectively [5,41], adapt better to the overwintering environment, and have better cold resistance [42,43,44].

### 4.5. Freezing Inside and Outside Plant Cells under Cold Stress

When plants experience cold stress, tissue freezing occurs, accompanied by the production of ice crystals, the location of which in plant tissues determines whether the cells die, or not [9]. Intracellular ice crystals cause death and extracellular ice crystals temporarily protect the cells [7,10,23]. After cold stress at −30 °C in the indoor experiment of *Pachira glabra Pasq.*, the leaves were wilted at room temperature and died after one day. This indicated that ice crystals caused by rapid freezing and thawing at too low a temperature had appeared in the cells of the plant tissues and had formed large ice crystals, which damaged cell structures (such as organelles and cell membranes), leading to plant death. This demonstrated the danger of intracellular ice crystals and rapid cellular freeze-thaw [5,6,7,8,22]. The persistence of ice content in *Lagerstroemia indica* L. and *Juniperus virginiana Linnaeus* over winter, and the return to normal growth in spring, demonstrate the persistence of extracellular ice crystals during winter. This suggests that with proper cold training, plant cells gradually adapt to dehydration caused by extracellular ice crystal formation [45], improving their ability to resist harsh survival conditions [5,23], thus providing a reference for plantation forestry care.

### 4.6. The Advantages and Disadvantages of the BVIC Sensor

Many researchers have applied image techniques, TDR sensors, ultrasonic acoustic emission analysis, and LVDT sensors to study plant freeze-thaw, and their performance has been demonstrated [6,16,31]. However, it is difficult to achieve real-time monitoring for image techniques, and although TDR sensors, ultrasonic acoustic emission analysis, and LVDT sensors are somewhat non- or minimally destructive, they are expensive. The BVIC sensor designed in this study is inexpensive (it costs approximately USD 20). Further, it is capable of dynamic real-time monitoring, it causes minimal damage to plants, it is applicable to fine branches for measurement, and is easy to use in practical application in agriculture and forestry, providing a technical means for the freeze-thaw monitoring and cold resistance assessment of plants. However, for some plants with a fast branch growth rate, BVIC sensors are not applicable at present; research into probes that can dynamically follow the branch diameter change, or establish a diameter-based compensation model, is a further problem to be solved in the future.

## 5. Conclusions

In this paper, we developed a miniature noninvasive intelligent sensor (the BVIC sensor) for detecting the volumetric ice content and the ice freeze-thaw rate in the branches of plants during the overwintering period by analyzing the impedance changes of the branches under cold stress. In a series of performance tests, the results showed that the feasible range of excitation frequency sources for impedance measurement circuits is from 100 MHz to 200 MHz, that the static and dynamic characteristics of the BVIC sensor met the requirements for practical use, and that ambient temperature and humidity or branch conductivity had little effect. Measurements were carried out on different plants, demonstrating the application of the method to multiple objectives. Monitoring *Pachira glabra Pasq.*, *Lagerstroemia indica* L. and *Juniperus virginiana Linnaeus*, indicated that the BVIC sensor can effectively monitor changes in the branch ice content. The differences in the volumetric ice content and the branch ice freeze-thaw rate of *Lagerstroemia indica* L. and *Juniperus virginiana Linnaeus* over winter demonstrated the BVIC sensor can effectively differentiate characteristics in plants with different cold resistance, and can be used as an effective monitoring tool for plant freeze-thaw studies. As well as image techniques, TDR sensors, ultrasonic acoustic emission analysis, and LVDT sensors, the BVIC sensor designed in this paper is inexpensive, can be applied to the installation of fine branches for measurement, and can be used either alone or integrated into other systems due to the internal collection of wireless communication modules. This study provides a theoretical basis for assessing embolism and plant cold resistance induced by cold stress, as well as a cost-effective tool for monitoring the freeze-thaw information of over wintering plants in the field. The method proposed in this paper can also be combined with plant assessment parameters [46,47,48] to study the prediction problem under time-series conditions [49,50,51] by coupled multiparameter characterization and parameter estimation algorithms [52,53] and applied to other engineering application systems [54,55,56]. For fast-growing plants, it is necessary to study dynamically adjustable measurement probes and complementary diameter-based methods. By deploying many sensors on a single tree, it is also possible to try to decipher the dynamics of the whole tree’s hardiness characteristics during the overwintering period, from the direction of temporal and spatial changes.

## Figures and Tables

**Table 1:** The GDJ-1000B internal environment temperature and humidity settings.

Temperature (°C)	Humidity (RH)		
−40	0%		
5	30%	60%	90%
35	30%	60%	90%
65	30%	60%	90%

**Table 2:** Fitting equations for the values of the volumetric moisture content of the branches and the output voltage values of the measurement circuit.

Tree Species	Fitting Equation	R^2^	*k*	*B*
*Juniperus virginiana Linnaeus*	θ=31.28×U−6.13	0.9845	31.28	−6.13
*Lagerstroemia indica* L.	θ=52.27×U−30.97	0.9803	52.27	−30.97
*Pachira glabra Pasq*.	θ=49.11×U−12.63	0.9892	49.11	−12.63

**Table 3:** The statistical analysis of the volatility of the ice freeze-thaw rate of the branch volume ice content during the overwintering period.

Tree Species	Mean Value	Standard Deviation	Minimum Value	Maximum Value
*Lagerstroemia indica* L.	0.01515	0.02256	0	0.87662
*Juniperus virginiana Linnaeus*	0.00294	0.00405	0	0.04446

## Data Availability

The data that support the findings of this study are available from the corresponding author upon reasonable request.
